# Systematic Review and Meta-analysis of AI-driven MRI Motion Artifact Detection and Correction

**Published:** 2025-09-05

**Authors:** Mojtaba Safari, Zach Eidex, Richard L.J. Qiu, Matthew Goette, Tonghe Wang, Xiaofeng Yang

**Affiliations:** a Department of Radiation Oncology and Winship Cancer Institute, Emory University, Atlanta, 30322, GA, USA; b Department of Medical Physics, Memorial Sloan Kettering Cancer Center, New York, 10065, NY, USA

**Keywords:** Deep learning, *k*-space, Motion correction, Motion detection, Artificial intelligence, Motion reduction

## Abstract

**Background:**

To systematically review and perform a meta-analysis of artificial intelligence (AI)-driven methods for detecting and correcting magnetic resonance imaging (MRI) motion artifacts, assessing current developments, effectiveness, challenges, and future research directions.

**Methods:**

A comprehensive systematic review and meta-analysis were conducted, focusing on deep learning (DL) approaches, particularly generative models, for the detection and correction of MRI motion artifacts. Quantitative data were extracted regarding utilized datasets, DL architectures, and performance metrics.

**Results:**

DL, particularly generative models, shows promise for reducing motion artifacts and improving image quality; however, limited generalizability, reliance on paired training data, and risk of visual distortions remain key challenges that motivate standardized datasets and reporting.

**Conclusions:**

AI-driven methods, particularly DL generative models, show significant potential for improving MRI image quality by effectively addressing motion artifacts. However, critical challenges must be addressed, including the need for comprehensive public datasets, standardized reporting protocols for artifact levels, and more advanced, adaptable DL techniques to reduce reliance on extensive paired datasets. Addressing these aspects could substantially enhance MRI diagnostic accuracy, reduce healthcare costs, and improve patient care outcomes.

## 1. Introduction

Magnetic resonance imaging (MRI) is a non-invasive medical imaging technique that provides high-resolution anatomical and functional information without using ionizing radiation. However, the acquisition of high-quality MR images often requires long scan times, which increases the likelihood of image degradation due to both voluntary and involuntary patient motion. Such motion can alter the static magnetic field $\left({\text{B}}_{0}\right)$ [1], induce susceptibility artifacts [2], affect spin history leading to signal loss [3], and cause inconsistencies in k-space sampling that violate Nyquist criteria [4]. Motion artifacts are among the most prevalent sources of image degradation in MRI [5]. These artifacts can also compromise the performance of post-processing tasks, including target tracking in MR-guided radiation therapy [6], image segmentation [7, 8], and machine learning-based classification [9].

Mitigating motion artifacts often necessitates repeating scans, which increases healthcare costs and contributes to patient discomfort. It is estimated that 15–20% of neuroimaging exams require repeat acquisitions, potentially incurring additional annual costs exceeding $300,000 per scanner [10, 11, 12]. Therefore, the development of effective motion detection and correction strategies is essential to ensure diagnostic accuracy and improve healthcare efficiency.

Approaches to motion mitigation are typically classified into two broad categories: prospective and retrospective correction. Prospective motion correction methods attempt to compensate for motion during image acquisition. These include external optical tracking systems with reflective markers [13], physiologic gating [14], and newer strategies using active NMR field probes [15, 16, 17]. Sequence-embedded navigators, such as PROMO and vNavs [18, 19], and navigator-free methods using dynamic image reconstruction [20] also fall within this category. Hybrid systems combining sensors and MR-based feedback have demonstrated increased robustness, particularly in challenging clinical scenarios [21, 22, 23].

Despite their utility, prospective methods face technical and logistical limitations. They often require hardware modifications, rigid coupling of sensors to the anatomy, or increased sequence complexity. These constraints can reduce their applicability in routine clinical settings and limit effectiveness for fast or non-rigid motion [4, 24].

In contrast, retrospective motion correction methods operate on data acquired during routine scans, without requiring additional hardware. Common techniques include rigid or non-rigid image registration, slice-to-volume reconstruction, and model-based reconstructions that jointly estimate both motion and image content [4, 25]. These approaches remain essential in clinical practice because they can handle residual artifacts that persist despite prospective correction and are more adaptable to a range of motion patterns.

Recent developments in deep learning (DL), particularly those adapted from computer vision, have shown great promise in enhancing both prospective and retrospective motion correction. For prospective applications, convolutional neural networks (CNNs) have been used to estimate motion from image navigators or k-space data with sub-second latency, enabling real-time feedback for acquisition control [26, 27, 28]. In retrospective settings, DL models can be trained to detect the presence and severity of motion artifacts, and to reconstruct motion-reduced or motion-free images using supervised, unsupervised, or unpaired learning strategies [29, 30]. Unlike conventional iterative algorithms, DL-based motion correction models can learn direct mappings between corrupted and clean images, often yielding improved perceptual quality and reduced reconstruction time. These models are particularly powerful when integrated with generative architectures such as GANs, cGANs, CycleGANs, and diffusion models, which can capture complex image priors and correct non-linear distortions.

Previous reviews have primarily offered narrative syntheses of DL approaches for mitigating motion artifacts in MRI, typically organizing methods by task or application. For instance, one review explored network training strategies for motion correction and introduced a simulation tool, but provided only a qualitative overview rather than a quantitative synthesis [31]. Another focused on rigid motion correction, summarizing model families and architectural choices, though without conducting a study-level meta-analysis [32]. A further review examined retrospective learning-based correction across acquisition sequences and reconstruction stages, but again did not include statistical aggregation of findings across studies [33]. Similarly, a separate work addressed DL for brain MRI motion correction, emphasizing algorithmic advances and illustrative examples rather than pooled quantitative evidence [34]. In contrast, this study integrates a systematic review with meta-analysis, extracting study level variables and quantifying temporal trends in datasets, designs, and image quality metrics, thereby extending prior narrative syntheses.

Given the growing diversity of DL-based motion detection (MoDe) and correction (MoCo) methods, a systematic review and meta-analysis are needed to summarize recent developments, identify common patterns, and evaluate the effectiveness of existing approaches. This study presents the first comprehensive meta-analysis of AI driven MRI motion artifact detection and correction methods, with an emphasis on generative models. Section 2 provides a review of DL model types, followed by motion simulation methods in Section 3, and an analysis of MoDe and MoCo approaches in Section 4. We then present the results of our meta-analysis in Section 6 and discuss future research directions in Section 7. Our key contributions are:

- A unified framework summarizing AI-driven MoDe and MoCo strategies (Figure 3).
- The first meta-analysis quantifying the performance of DL-based motion artifact detection and correction models.
  - Assessment of image quality metrics (e.g., PSNR, SSIM), and comparison across studies.
  - Temporal trends in dataset usage (public vs. institutional), model components, and evaluation metrics.
  - Evaluation of hyperparameters such as learning rate, loss functions, and implementation frameworks.

## 2. Deep learning

DL algorithms, a subset of machine learning, are particularly effective at modeling complex and nonlinear relationships, especially in computer vision and medical imaging tasks [35]. In medical imaging, DL models have been successfully applied to a wide range of applications, including anatomical segmentation [36, 37, 38], image registration [39], image enhancement [40, 41], super-resolution [42, 43, 44], modality synthesis [45, 46, 47], and disease classification [48, 49, 50].

The trainable parameters of a network are optimized to generate outputs that closely match the target outputs. In the context of motion artifact correction, the network parameters are finely adjusted to transform input motion-corrupted images into motion-free images. On the other hand, the MoDe network parameters are optimized to enhance the models in predicting the presence of motion artifacts and their severity level. This refinement process is known as training, during which the network adjusts its parameters based on the difference between the prediction and target ground truth, such as motion-free images for MoCo and motion severity levels for MoDe. The loss function, which measures the discrepancy between actual and desired outputs, guides the network in updating its parameters to model optimal DL-based MoDe and MoCo processes effectively.

Among the various types of DL models, deep generative models have the potential to revolutionize the field by enabling advanced data synthesis and representation learning. The ability of generative models to synthesize realistic and diverse motion-corrupted and motion-free data, as well as to capture complex distributions of motion patterns, will be crucial for advancing the robustness and generalizability of future motion correction and detection methods.

### 2.1. Deep generative models for motion artifact correction

Generative models are a class of DL methods designed to learn the underlying distribution of data so they can generate new samples that resemble the original dataset [51, 52]. Unlike discriminative models, which learn decision boundaries to classify or distinguish between data points, generative models focus on capturing data structure and variability. Key families include variational autoencoders [53], generative adversarial networks (GANs) [52], and diffusion models [54], each of which employs different mechanisms to approximate complex data distributions. In medical imaging, these models are especially relevant because they can synthesize realistic examples, augment limited datasets, and improve motion correction and detection by modeling non-linear motion-related distortions [55].

#### 2.1.1. Generative adversarial network

GANs [52] have revolutionized the field of medical image processing, particularly in tasks such as image synthesis [56], segmentation [57], denoising [58], and harmonization [59]. Beyond these specific applications, early surveys and foundational works have highlighted the versatility of GANs in medical imaging, including their use for cross-modality translation, data augmentation, and domain adaptation [55, 45].

GANs consist of two networks, including a generator and a discriminator, that are trained simultaneously in an adversarial framework to generate realistic data from input noise. The generator receives an input $z∼q\left(z\right)$ and transforms it to $z^{*}$ using a network $G_{\kappa }$ with parameter κ. Simultaneously, the discriminator $D_{\vartheta }$ with parameters ϑ distinguish generated realistic image $z^{*}$ from real image $x∼p^{*}\left(x\right)$. The discriminator objective is to minimize the Jensen-Shannon divergence between $q_{\kappa }$ (the likelihood function of the generated data $z^{*}$) and $p^{*}$ (the distribution of real data x) [51], which leads to the following loss function:

(1) $$\underset{\kappa }{\min}\;\underset{\vartheta }{\max}\;E_{x∼p^{*}\left(x\right)}\left[\log D_{\vartheta }\left(x\right)\right]+E_{z∼q\left(z\right)}\left[1-\log D_{\vartheta }\left(G_{\kappa }\left(z\right)\right)\right]$$

GANs have been applied directly to remove brain motion artifacts [60, 61, 62], and to define an adversarial regularizer to enhance the model performance [63].

However, GANs face limitations in controlling the traits of generated outputs and are prone to mode collapse, where the model produces a limited variety of outputs. Conditional GANs (cGANs) address these issues by introducing conditional inputs to guide the generation process and allow for specific control over the characteristics of the outputs. This approach not only provides greater control but also helps mitigate mode collapse by encouraging the generation of more diverse and varied samples based on the given conditions [64]. Thus, several MoCo models have leveraged cGANs to reduce or remove motion artifacts [65, 66, 67, 68]. cGANs also have some limitations, namely the need for paired training data, which CycleGANs can help address. While cGANs require paired examples to learn the mapping from input to output (e.g., translating a motion-corrupted image to its motion-free counterpart), CycleGANs can learn this mapping using unpaired data. CycleGANs achieve this by introducing a cycle consistency loss, ensuring that an image translated from domain A to domain B and back to domain A remains unchanged, thus enabling effective domain translation without the need for paired datasets [69]. Eliminating the image pair requirement makes the CycleGAN training framework attractive for unsupervised MoCo models [70, 71, 72, 73].

#### 2.1.2. Denoising diffusion probabilistic model

**Figure 1:**
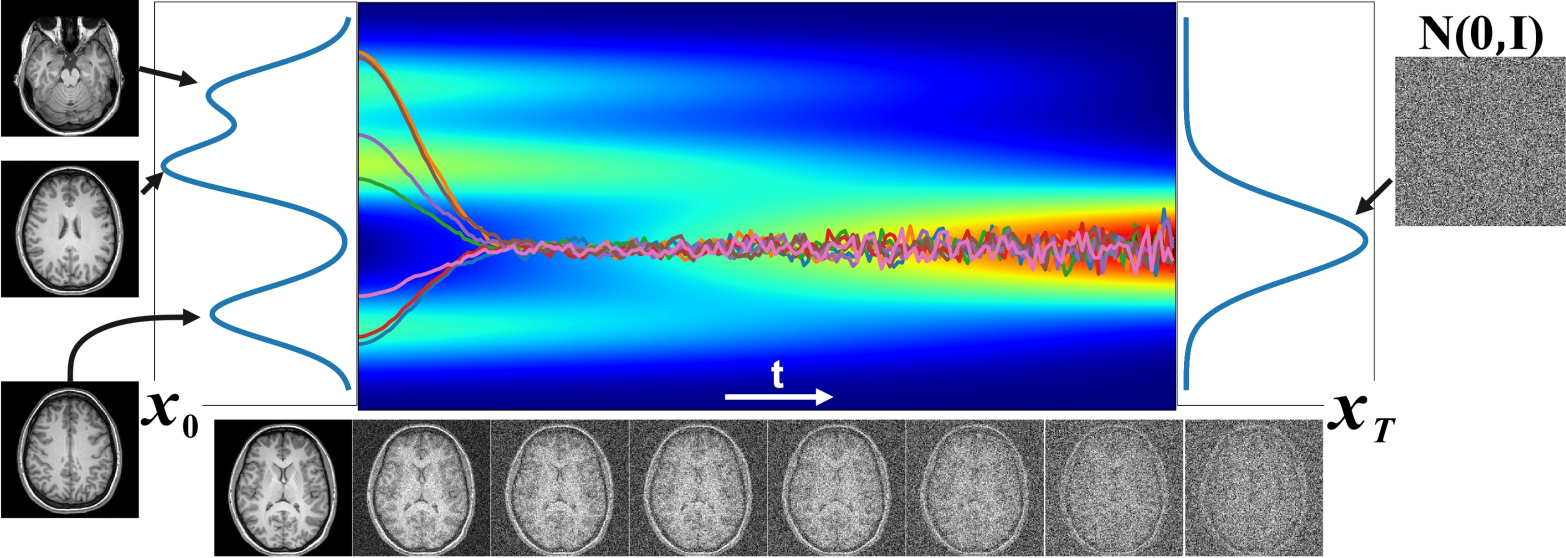
Trajectory plot illustrating the evolution of samples from a three-mode Gaussian mixture distribution as they transition to a standard normal distribution $𝓝\left(0,I\right)$. Here we simulated the Gaussian mixture as $\sum_{i=1}^{3}\omega_{i}𝓝\left(\mu_{i},\sigma_{i}\right)$ with $\mu ={\left[-2,2,4\right]}^{T}$ and $\sigma ={\left[0.5,0.5,0.5\right]}^{T}$ and forward diffusion step was performed over T=1000 steps.

The denoising diffusion probabilistic model (DDPM) is a generative model aimed at approximating complex intractable distributions with simple and tractable distributions, such as the normal Gaussian distribution [74]. The DDPM consists of two processes: a forward process and a reverse process. The forward process injects controlled Gaussian noise into the input image over a large number of steps, denoted as T, until the image is transformed into normal Gaussian noise. This process heavily utilizes the first-order Markov process, which markedly reduces the computational time. On the other hand, the reverse process trains a deep neural network (DNN) to recover input image from normal Gaussian noise over T steps as shown in Figure 1 by minimizing the following loss function:

(2) $$\underset{\kappa }{\arg\min}{\left\|\epsilon_{0}-{\hat{\epsilon }}_{\kappa }\left(x_{t},t\right)\right\|}_{2}^{2}$$

where the neural network $\hat{\epsilon }$ with parameter κ learns to predict the source noise $\epsilon_{0}∼𝓝\left(\epsilon \left|0,I\right.\right)$ that determines noisy data $x_{t}$ in step t from noise-free image $x_{0}$.

Although the DDPM models were initially developed to generate images from normal Gaussian noise, they have also been used to remove motion artifacts from anatomical brain images [75, 76].

#### 2.1.3. Risks of hallucinations in generative models

A critical challenge in applying generative models to medical imaging is the risk of hallucinations, where networks synthesize artificial features that appear realistic but do not correspond to the underlying anatomy. Such hallucinations can arise when the generator overfits to distribution-matching objectives or when training datasets are limited or biased. Although visually plausible, these artifacts may introduce false structures or remove clinically relevant features, which poses a significant risk of misdiagnosis in clinical practice [77, 78].

Concrete examples of hallucinations have been demonstrated in prior work. Kazeminia et al. [79] illustrated spurious details in GAN-generated medical images, while Cohen et al. [80] showed that distribution matching losses in image translation can hallucinate features, and Bhadra et al. [81] analyzed hallucinations in tomographic image reconstruction. Reviews of GAN applications in medical imaging have also highlighted hallucination risks as a major limitation for clinical deployment [55].

To mitigate these risks, strategies such as uncertainty quantification [82], physics-informed training objectives [83], and adversarial regularization [84] have been proposed. However, further research is needed to develop standardized evaluation protocols that can reliably identify and quantify hallucinations in generated images. Addressing this limitation is essential to ensure that generative models can be safely integrated into clinical workflows.

### 2.2. Supervised and unsupervised training frameworks

Training MoCo and MoDe models can be broadly categorized into two approaches: supervised and unsupervised. The supervised approach relies on the availability of paired datasets where each motion-corrupted image has a corresponding motion-free counterpart. This enables MoCo models to learn the transformation from the input (motion-corrupted images) to the target (motion-free images). Similarly, in the context of MoDe, paired datasets are essential for accurately predicting the presence and severity of motion artifacts.

However, acquiring large, high-quality paired datasets is both challenging and costly, presenting a significant obstacle to the widespread implementation of supervised frameworks. To address this limitation, alternative frameworks that reduce the dependency on paired data have been developed. These alternatives can be divided into two categories: unpaired and unsupervised methods. Unpaired methods utilize motion-free datasets from the same MRI sequence to remove motion artifacts from similar but unpaired MRI sequences [70, 71, 73]. Despite their potential, these methods face practical challenges, such as the difficulty of acquiring motion-free images from different patients, which can raise clinical and privacy concerns, particularly regarding data sharing outside of hospital systems.

To address these limitations, unsupervised methods have been developed that eliminate the need for unpaired datasets. These methods leverage transfer learning techniques [85] and auxiliary information from other MRI sequences [72] to achieve effective motion correction and detection without the requirements of the previous approaches. These advancements offer a promising direction for reducing the reliance on large-scale datasets and enhancing the feasibility of AI-driven motion artifact correction and detection in clinical settings.

## 3. Motion simulation

The supervised MoCo and MoDe models require a large dataset containing paired motion-free and motion-corrupted images, which is time-consuming and expensive to acquire [86]. Thus, generating *in-silico* motion-corrupted images can avoid the paired data requirement. In addition, motion simulation can serve as an augmentation technique to improve the generalization of DL models in tasks such as segmentation [87, 88].

**Figure 2:**
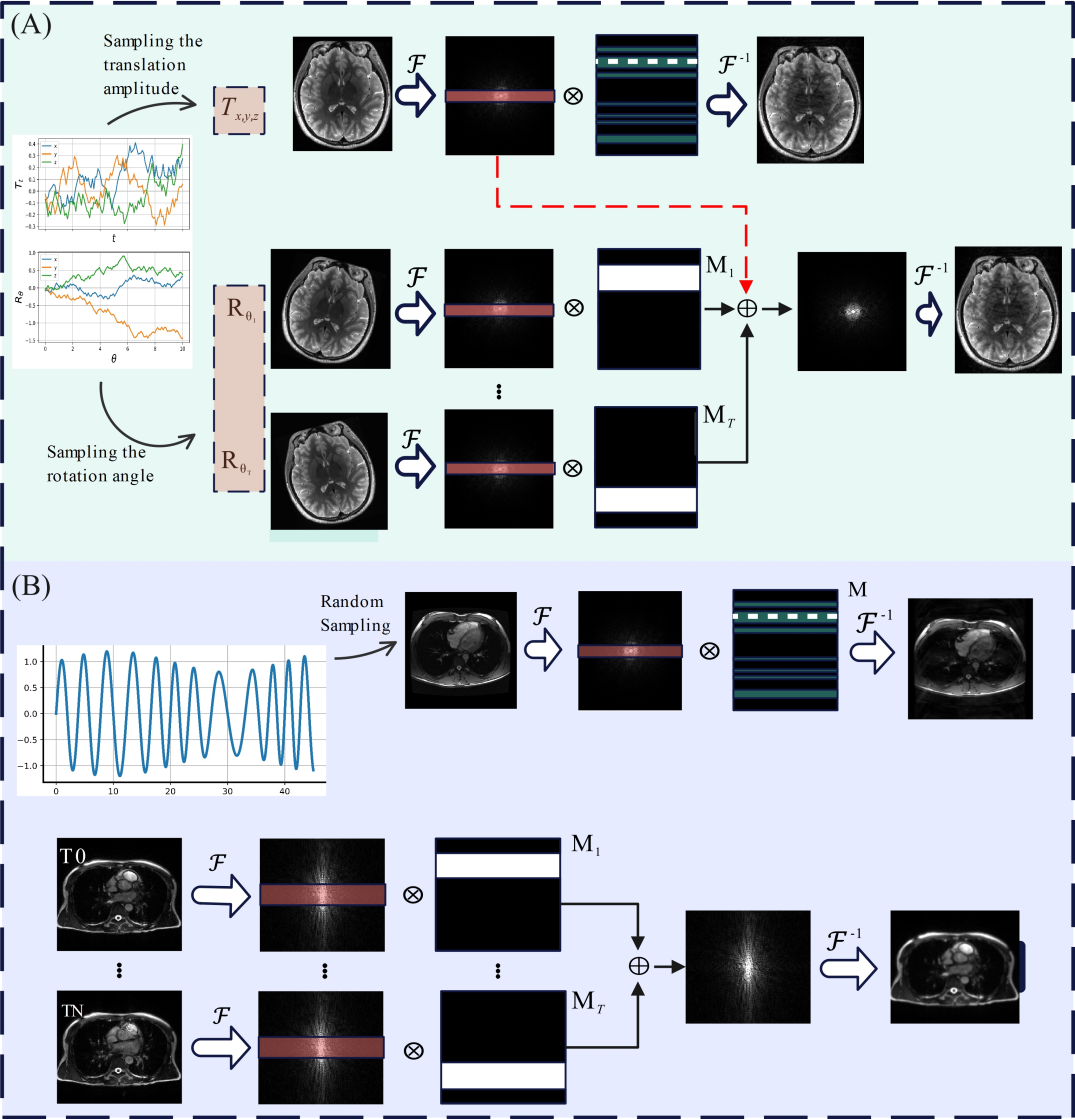
Motion simulation methods are illustrated. (A) The abrupt brain movement involves rigid motion and rotation, simulated by randomly sampling translation $T_{x,y,z}$ and rotation $R_{\theta_{i}}$. (B) The abdominal and cardiovascular coherent movement simulation introduces blurring to the images. Motion simulation methods for static and dynamic MR images are shown. The center of k-space data, shown by red slabs, is excluded from modifications.

Patient motion induces phase shift in k-space data, which results in discrete ghost artifacts along the phase encode direction when the motion is periodic in nature, respiratory and cardiac motion, and diffuse image noise when the motion is aperiodic, like peristalsis. Thus, realistic motion-corrupted images can be generated by randomly manipulating k-space lines along the phase encoding (PE) and slab encoding directions, which are markedly slower to acquire than the lines along a readout direction [31]. This section covers the simulation of abrupt and coherent motion artifacts that are apparent for brain, cardiac, and abdominal MR images, respectively. The motion simulation techniques are illustrated in Figure 2.

### 3.1. Head movement

Rigid head movement is modeled using rotation and translation matrices. Translation movement is simulated by randomly shifting the k-space lines along the PE direction $k_{y}$ as follows:

(3) $$Y_{\text{distorted}}\left(k_{x},k_{y}\right)=\left\{\begin{array}{cc} Y\left(k_{x},k_{y}\right)e^{-j\phi \left(k_{y}\right)} & k_{y}\in 𝓓\\ Y\left(k_{x},k_{y}\right) & \text{otherwise}, \end{array}\right.$$

where Y and $Y_{\text{distorted}}$ are motion-free and motion-corrupted k-space images. The distortion phase $\phi \left(k_{y}\right)$ controls the distortion level, 𝓓 is the randomly selected k-space PE lines, and $k_{y}$ is the distorted line along PE, as shown by a dashed white line in Figure 2A. The translations $T_{x,y,z}$ are randomly sampled to simulate the distortion phase $\phi \left(k_{y}\right)$ given below:

(4) $$\phi \left(k_{y}\right)=\left\{\begin{array}{cc} k_{y}\Delta & \left|k_{y}\right|>k_{0}\\ 0 & \text{otherwise}, \end{array}\right.$$

where Δ controls the motion artifact level and $k_{0}$ is a delay time of the phase error due to centric k-space filling. The term $k_{0}$ refers to a threshold or specific point in k-space, particularly in the PE direction, where the phase errors start to become significant. Before this point, the phase errors are either minimal or do not contribute substantially to the artifacts [89].

Furthermore, abrupt rotational movements are generally simulated in both k-space and the image domain. The rotation $R_{\theta_{i}}$ values are randomly sampled to rotate the images, and their corresponding k-space data are sub-sampled using random masks $M_{i}$. Finally, the sub-sampled k-space data are added to the k-space data of motion-free images (red dashed line in Figure 2A) to generate motion-corrupted data. This process is shown in Figure 2A (lower panel), where the center of k-space data was excluded from motion simulation.

### 3.2. Abdominal and cardiovascular movement: coherent motion

Coherent motion typically occurs due to patients’ involuntary movement, which can introduce significant blurring and ghosting artifacts. A random distortion phase $\phi \left(k_{y}\right)$ can be sampled from a quasi-sinusoidal as shown in Figure 2B to simulate motion artifacts for static MR images. The coherent distortion phase is as follows:

(5) $$\phi \left(k_{y}\right)=\left\{\begin{array}{cc} k_{y}\Delta \sin\left(\alpha k_{y}+\beta \right) & \left|k_{y}\right|>k_{0}\\ 0 & \text{otherwise}, \end{array}\right.$$

where α and β are frequency and phase shift constants and Δ is a random distortion level.

To simulate coherent motion artifacts in dynamic MR images, random masks $M_{i}$ are used to sub-sample the images acquired at different times. The sub-sampled k-space lines are then combined to generate motion-corrupted images. This method also excludes the center of the k-space data to preserve the low-frequency content of the images.

## 4. Motion Correction and Detection Models

**Figure 3:**
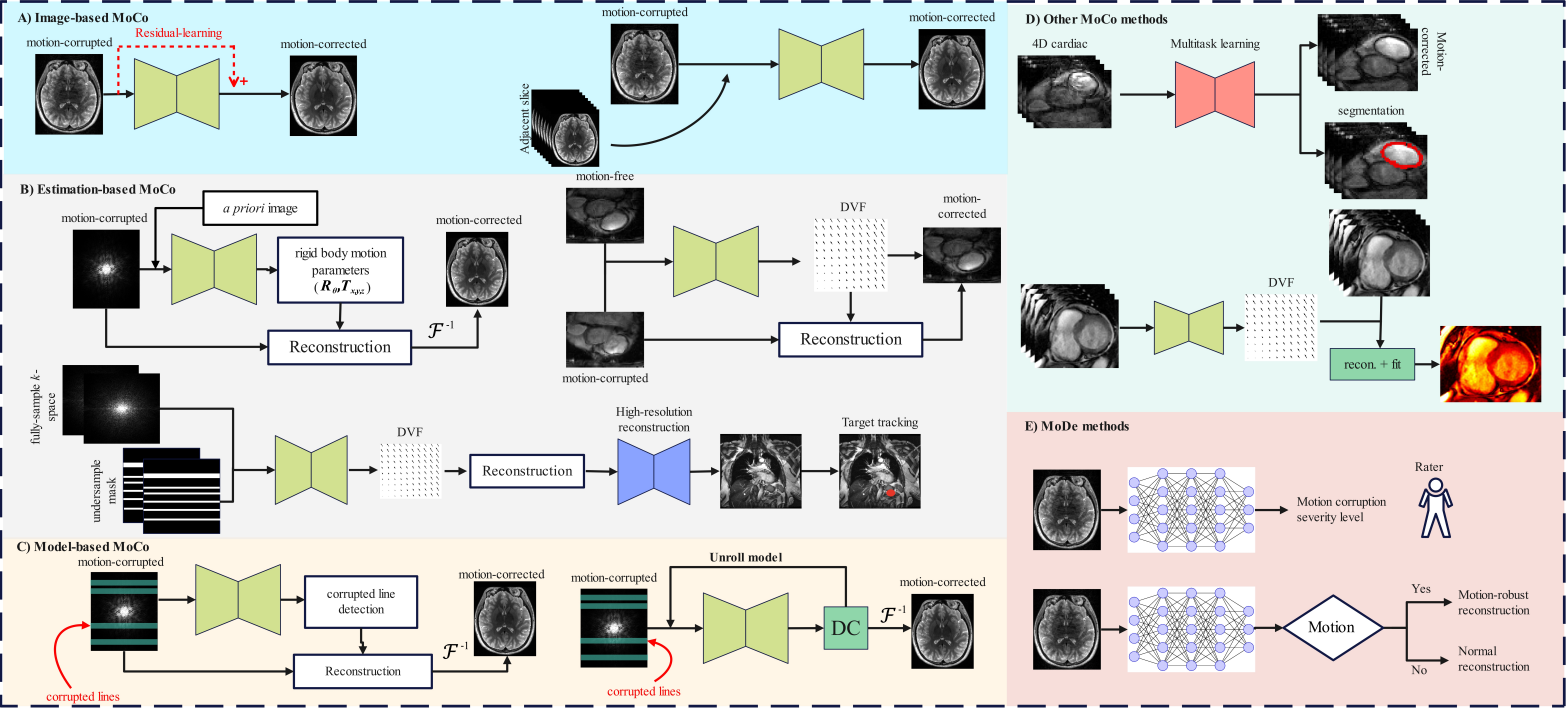
Illustration of the overall DL-based MoCo and MoDe models. (A) left depicts an image-based training method that uses motion-corrupted and motion-free images, with an alternative approach focusing on reconstructing residual errors to generate motion-free images (red dashed line). (A) right shows an enhancement to this method using auxiliary images (data) to enhance the previous method. (B) presents motion estimation-based models, where a DNN in the left panel estimates the rigid motion parameters and another DNN in the right panel reconstructs the deformation vector field (DVF). This approach also includes the application of an DNN for DVF reconstruction from under-sampled k-space, crucial for real-time target tracking in image-guided radiation therapy.(C) illustrates model-based methods trained on k-space data. The left panel uses an DNN to estimate the amount of rigid motion, which is then used to reconstruct motion-free images iteratively. The right panel illustrates a method that unrolls the model into two modules, a denoiser DNN and data consistency module for motion artifact correction. The estimated motion parameters are used to correct the motion artifacts. (D) illustrates other types of motion-correction methods, including multi-task learning and quantitative MRI. (E) represent motion detection method, which are used either to detect motion artifacts or to select appropriate downstream tasks, such as motion-robust image reconstruction.

This section outlines the MoDe and MoCo methods. MoCo techniques are broadly classified into four categories: image-based, estimation-based, model-based, and other techniques where MoCo models serve as auxiliary tasks. MoDe methods are broadly categorized into two groups: models that predict the presence and severity of motion artifacts, and models designed to select the most appropriate downstream method for specific tasks, such as image reconstruction. These categories are illustrated in Figure 3.

### 4.1. Image-based MoCo

An image-based correction technique models the DL-based MoCo directly in the image domain, as illustrated in Figure 3A. The motion artifact model is represented by:

(6) $$y={𝓕}^{-1}𝓐𝓕x,$$

where $x\in {\mathbb{R}}^{N_{x}\times N_{y}\times N_{z}}$ is a motion-free image with $N_{i}$ for $i=\left\{x,y,z\right\}$ voxels, 𝓕 is the Fourier transform, 𝓐 is a motion-corruption model in k-space, ${𝓕}^{-1}$ is the inverse Fourier transform, and $y\in {\mathbb{R}}^{N_{x}\times N_{y}\times N_{z}}$ is the motion-corrupted images. We assumed the images were real-valued for simplicity. Image-based correction techniques address this inverse problem directly, mapping from motion-corrupted images $\left(y\right)$ to motion-free images $\left(x\right)$.

One advantage of this approach is its capacity to incorporate new advancements seamlessly and models from various DL domains. For instance, the U-net model was initially designed for segmentation tasks but was easily repurposed to produce motion-free images with minimal adjustments. Similarly, generative models such as GANs [52] and DDPM [54] were initially developed to generate images from noise and have since proven useful in mitigating motion artifacts [30, 71, 67, 68, 90, 91, 76, 92, 93, 94, 95, 96].

However, a potential limitation of this approach is its reliance on large volumes of data, as well as the risk of introducing false visual perceptions, particularly in cases with severe motion artifacts. To address these challenges, image-based MoCo techniques have been developed that utilize auxiliary images or perform corrections across multiple adjacent image slices. This additional information guides the training process, as depicted in Figure 3A (right panel) [97, 98, 99, 100].

#### 4.1.1. Residual learning

Our literature review identified three studies that aimed to predict the difference between motion-corrupted and motion-free images (residual errors) [101, 89, 102]. The concept of residual learning suggests that networks produce improved motion-free images by predicting the residual errors with smaller variations than motion-free images. Furthermore, this method may bypass the constraint of preserving the soft-tissue contrast of motion-free images, potentially leading to the generation of sharper images. This approach is shown with a red dashed line in Figure 3A (left panel).

### 4.2. Estimation-based MoCo

Estimation-based approaches, which involve estimating motion parameters using a DNN, can be categorized into two main groups: rigid-motion artifact estimation and deformable motion-artifact estimation methods. These methods can also serve as auxiliary tasks for reconstructing high-resolution images from down-sampled, motion-corrupted MRI data, thereby accelerating the imaging process - an essential factor for target tracking during radiation therapy. The general mathematical formulation for rigid-body motion artifact modeling is as follows [103]:

(7) $$k_{\text{dist}}^{n}=\sum_{j=1}^{J}\underset{E_{\theta j}}{\underset{︸}{M^{j}𝓕C^{n}T_{x,y,z}^{j}R_{\theta }^{j}}}x,$$

where $k_{\text{dist}}^{n}\in {\mathbb{C}}^{N_{x}\times N_{y}\times N_{z}}$ motion distorted k-space for a given coil n, j represents the number of time steps that rigid distortion occurs, $M^{j}$ is a binary mask to select the corresponding k-space line in step j, $C^{n}$ is a sensitivity map of coil n, $T_{x,y,z}^{j}$ is a translation matrix, and $R_{\theta }^{j}$ is a rotation matrix. In rigid-body motion artifacts, such as those encountered in brain imaging, a DNN is trained to estimate rotation and translation parameters at each time step $E_{\theta j}$, with $\theta^{j}\in {\mathbb{R}}^{\text{DOF}}$. In 3D motion, the degree of freedom (DOF) is six (three translations and three rotations), while in the case of 2D in-plane rigid-body motion, only three parameters are needed (two translations and one in-plane rotation).

These parameters are then used in an iterative correction process where the initial motion estimates are used to correct the k-space data to generate a preliminary image, which may still contain residual motion artifacts. The process iterates by refining the motion estimates based on the partially corrected image, reapplying these refined corrections to the k-space data, and reconstructing the image again. This loop continues until the motion estimates converge, resulting in a final high-resolution image with minimal motion artifacts. The iterative approach ensures that even complex and non-linear motion is corrected accurately, leading to a clear and reliable final MR image [104, 105, 106].

Considering that multiple MRI sequences are acquired subsequently in an single imaging session, this technique can be extended to use auxiliary input images–possibly from different sequences–to guide the training process of estimating θ and correct for rigid motion artifacts [107, 108].

It is noteworthy that the mentioned method is particularly effective for correcting rigid motion artifact. To extend this method to the regions with non-rigid deformations voxel-wise deformation vector fields (DVFs) must be estimated using a DNN, as shown in Figure 3B (right panel). The predicted DVF is then used to correct the non-rigid motion artifacts [109, 110]. Furthermore, this technique can be combined with the acceleration algorithms to compensate for involuntary abdominal motions [111, 112, 27] and super-resolution [113, 114].

### 4.3. Model-based MoCo

Given the limitations of image-based MoCo models, including the requirement for large datasets and the potential for hallucinations, model-based MoCo models incorporate a data acquisition model to remove motion artifacts. This approach can be categorized into two techniques corrupted k-space line and methods that unroll the training process (see Figure 3B right panel).

In the former, a DNN is employed to detect k-space lines affected by motion. The prediction results are then integrated into an iterative reconstruction process to reduce motion artifacts in the final images [115, 116]. An alternative approach leverages a combination of DL models and k-space analysis to identify motion-affected lines. This method filters the motion-corrupted images using a convolutional neural network, compares the filtered k-space data with the original motion-corrupted k-space, and reconstructs the final image using only the unaffected k-space lines with compressed sensing, effectively mitigating motion artifacts [117, 118].

The latter technique splits the models into a denoising network and data consistency layer. The denoiser reconstructs a first estimation of motion-free images to solve the data fidelity by a gradient descent method in an iterative fashion, as follows [119]:

(8) $$x_{t+1}=x_{t}-2\lambda {𝓐}^{H}\left(y-𝓐x_{t}\right)$$

where 𝓐=𝓕C is the encoding matrix, λ is the learning rate to balance between the current estimation and the updated step size, ${𝓐}^{H}$ is Hermitian transform of 𝓐, and $x_{t}$ is the image at iteration t, with $x_{0}$ being the output from the denoiser [119, 120].

### 4.4. Other MoCo methods

MoCo models can be modified to perform several tasks simultaneously since multi-task DL models tend to generalize better, and auxiliary tasks can enhance the model’s performance on the primary task due to potential correlations between the tasks [121]. This adaptability allows for the integration of MoCo techniques with other DL-based approaches, such as segmentation [6, 122, 123] or quantitative MRI reconstruction [124, 125], resulting in multi-task models that achieve comprehensive results while maintaining high performance across each task.

### 4.5. Image-based motion detection

Image-based motion detection techniques focus on predicting motion artifacts directly using the image domain. This approach offers the advantage of incorporating advances from other domains of DL without requiring significant changes. For example, models like 3D-CNN [126], VGG16 [127], EfficientNet [128], AlexNet [129], and ResNet [130] have been adapted effectively to classify natural images. These models were also used to predict the presence and severity of motion artifacts in cardiothoracic and brain regions [131, 132].

Furthermore, MRI reconstruction techniques have been developed to mitigate the impact of patient movement during imaging [133, 134]. Inspired by this, DL-based methods have been proposed to assess the severity of motion artifacts and to adapt the reconstruction process accordingly. This approach involves predicting the level of motion artifact severity from undersampled MR images, which can then be used to accelerate acquisition [135] and to determine whether a standard or a motion-robust reconstruction method should be employed [10].

In line with this, a DL-based technique using a hierarchical convolutional neural network has been developed to reduce residual motion effects in diffusion MRI data [136]. This method selectively rejects only the most severely motion-corrupted data, while retaining the remaining data for accurate diffusion parameter estimation, thus enhancing the robustness of MRI reconstructions even in the presence of significant motion artifacts.

## 5. Meta-analysis method

### 5.1. Data collection

We collected literature across PubMed to evaluate quantitatively the trend of DL-based MoCo and MoDe models. The search utilized the keywords: “MRI” or “magnetic resonance imaging” and “motion” and “deep learning.” We used the keyword “motion” in lieu of “motion correction” or “motion reduction” to broaden the search range. The first search was carried out in May 2024 but we have included new published papers after that date. We exported the references of the matched studies as a (PubMed) .txt file and imported them into Zotero Desktop.

#### 1. Initial filtering:.

Initially, 449 studies were selected that match the keywords. After removing 91 duplicates and irrelevant studies, 358 non-duplicated studies remained.

#### 2. Title and Abstract screening:.

We reviewed the titles to determine the relevance of each study and screened out those that did not contain the keywords “MRI,” “motion,” “deep learning,” and “reduction”. One reviewer with previous experience in DL and MRI motion correction examined the abstract section to determine the relevant studies. In case the Abstract section was generic, the Introduction section was examined. This process left us with 121 studies, which advanced to the next data collection stage.

#### 3. Full-text screening.

**Figure 4:**
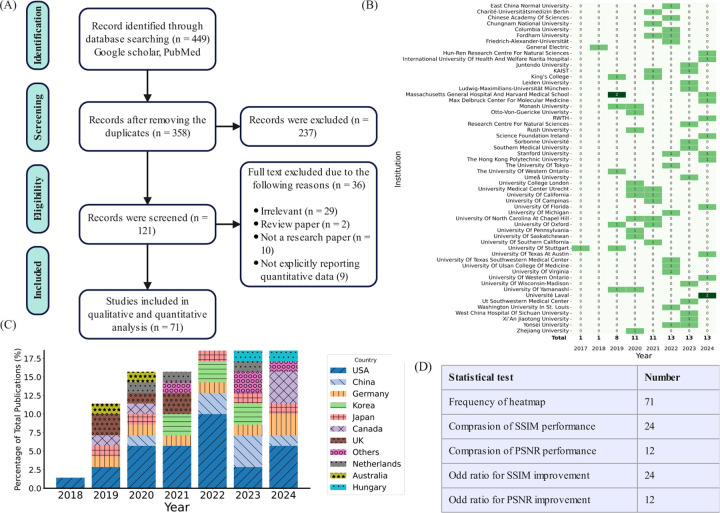
Information of the reviewed studies. (A) The preferred reporting items for systematic reviews and meta-analyses (PRISMA) flowchart of this meta-analysis study [138]. (B) Number of publications by research institute across years, illustrating the major contributing institutions and highlighting leading research hubs in DL-based MoCo and MoDe. (C) Number of publications by country, showing the global distribution and growth of research activity, which provides context on regional contributions and opportunities for collaboration. (D) Number of studies included for each type of statistical analysis performed in this review.

We read the full text and inspected the results. Finally, 71 studies were included. The entire process is illustrated in Figure 4A.

#### 4. Data collection:.

We modified the criteria given in the checklist for artificial intelligence in medical imaging (CLAIM) criteria [137] (see Table 1) to extract data from the studies. The modified table includes data critical for MoCo and MoDe studies, such as motion simulation methods, motion artifact levels, and quantitative metrics, which are not necessarily required for general-purpose medical image analysis.

### 5.2. Data analysis

#### 1. Statistical rationale and analysis plan:.

The study was the unit of analysis. Each patient or volunteer was counted once; image slices were not treated as independent samples. When a study reported development, evaluation, and testing sets, we combined evaluation with testing for summaries because both reflect out of sample performance. We distinguished 2D and 3D acquisitions due to differences in acquisition time and spatial resolution.

Reporting of dispersion was inconsistent across studies. Because many papers did not provide standard deviations or confidence intervals for PSNR and SSIM, we used two complementary strategies. First, for descriptive cross study comparison we transformed each metric to a dimensionless quantity, the multiple of the mean (MoM), defined as the study value divided by the corpus mean for that metric. MoM places heterogeneous scales on a common footing without assuming equal variances. We compared MoM distributions across publication years with nonparametric tests. Second, when a subset of studies reported dispersion, we ran sensitivity checks with meta regression on raw values and reached the same qualitative conclusions.

Temporal trend analyses served two goals. For continuous counts or proportions over time we report Pearson r and Spearman ρ to capture linear and monotonic associations. For year wise comparisons of sample sizes and MoM we used the Kruskal Wallis test because group sizes are unequal and normality is not guaranteed. To evaluate whether image quality improved over time, we fit ordinary least squares meta regression models with publication year as the predictor and PSNR or SSIM as the response, and we report heteroskedasticity robust standard errors.

#### 2. Development trend:.

We assessed temporal trends in publication volume and other continuous summaries using Pearson correlation coefficients and also report Spearman correlations to capture monotonic relations that may not be linear. Year wise comparisons of numerical variables, such as training and testing sample sizes, were performed with the Kruskal Wallis test because group sizes are unequal and normality is not guaranteed. All analyses used scipy.stats 1.13.1 [139] and statsmodels.api 0.15.0 (https://github.com/statsmodels/statsmodels/) in Python 3.10.14.

In our analysis, each patient or volunteer was treated as an individual sample rather than considering image slices. When a study divided data into training, evaluation, and testing sets, we incorporated the evaluation dataset into the testing dataset size. Additionally, we distinguished between 3D MRI and 2D MRI images, such as T_1_-weighted (T_1_w) versus 3D T_1_w, due to differences in acquisition time and spatial resolution.

#### 3. Performance:.

We divided each performance metric by its average across all studies to obtain the multiple of the mean (MoM), which reflects the relative improvement of models over time. We reported the average MoM values when models were tested under different motion artifact levels. The Kruskal-Wallis test was used to calculate *p*-values for MoM across years.

## 6. Meta-analysis results

**Figure 5:**
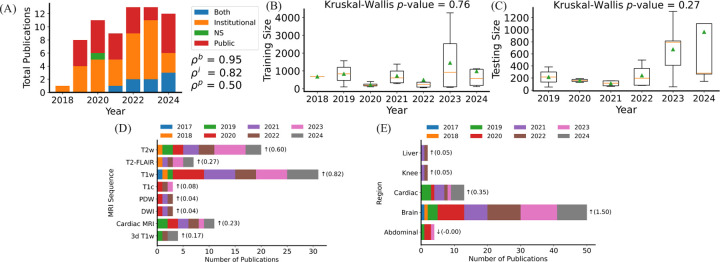
Dataset information from the reviewed studies. (A) The total number of publications using institutional, public, and both types of datasets over time is illustrated. The Pearson correlation between the number of publication uses public, private, and both with time are $\rho^{p}$, $\rho^{i}$, and $\rho^{b}$, respectively. Abbreviation: NS: Not specified. (B) and (C) Training and testing sample size are illustrated over time. The Kruskal-Wallis *p*-values were reported for each box plot. (D) MRI sequences used by the studies grouped by year of publication are illustrated. Abbreviations: FLAIR: fluid-attenuated inversion recovery, T1c: postcontrast T_1_-weighted, PD: proton density, DWI: diffusion-weighted imaging. (E) Anatomical region of the images used by the studies. The arrow shows the usage trends over time and the numbers inside parenthesis represents the slope value obtained from linear regression represents the rate of change in the number of publications per unit change in the year.

Out of the 71 reviewed studies that met our meta-criteria shown in Figure 4A and 4D, we observed increasing number of publications between 2018 and 2024, with the USA contributing the most to the research studies (see Figure 5C). Massachusetts General Hospital and Harvard Medical School was identified as the institution with the highest number of publications as shown in Figure 4B.

### 6.1. Dataset characteristic

Training and evaluation of MoCo and MoDe models require ground truth data. For MoDe, ground truth typically consists of quality scores (e.g., between zero and five) assigned by radiologists, because simulated datasets might not capture the full diversity of real motion artifacts and scanner-dependent variations. In contrast, MoCo datasets can often be generated by simulation methods (see Section 3), which eliminates the necessity for paired motion-free and motion-corrupted acquisitions.

In terms of dataset usage, 49.3% of the studies utilized institutional datasets exclusively, 37.3% used public datasets exclusively, and 11.9% used both institutional and public datasets. The most frequently utilized public datasets were fastMRI [140] and movement-related artifacts (MR-ART) [141], followed by the Human Connectome Project (HCP), UK Biobank [142], and IXI (https://brain-development.org/ixi-dataset/) datasets. These datasets and the corresponding studies are listed in Table 2. There is a noticeable increase in the use of public datasets in 2024, as depicted in Figure 5A. The Pearson correlation coefficients for the use of institutional, public, and combined datasets are 0.82, 0.50, and 0.95, respectively. These values show the strength of the linear relationship between time and the number of studies using each dataset type. The institutional datasets exhibit a robust positive correlation of 0.82, indicating a significant increase in the number of studies utilizing institutional datasets over time. Public datasets show a moderate positive correlation of 0.50, suggesting a steady but less pronounced growth in the number of studies using public datasets over time. In contrast, combined datasets have a robust positive correlation of 0.95, indicating a nearly perfect increase in the number of studies integrating institutional and public datasets over time. These correlations highlight that while the use of institutional datasets alone is growing robustly, there is an increasing trend towards integrating both institutional and public datasets, reflecting a shift towards more comprehensive and multifaceted data usage in research studies over the observed timeframe.

On average, studies used 65% and 35% of data for training and evaluation. The Kruskal-Wallis tests indicate the train and evaluation cohort populations did not significantly change overtime with p-values of 0.57 and 0.76 , respectively (see Figure 5B and C). The big jumps in cohort population in 2023 and 2024 are due to the MoDe studies that utilized several large public datasets. To increase the data population, eight studies employed data augmentations including mainly random translation, flip, and rotation. One study used methods such as adding random noise and bias fields to further increase the data population [111]. Patch-based model training was utilized in two studies to increase data samples [101, 106].

Among the MRI sequences, T_1_w, T_1_c, and CMR were the most frequently used, with 30, 30, and 10 studies, respectively, reporting their use (see Figure 5D). Linear regression analysis indicated an increasing trend in their usage, as shown by the arrows. Among the imaging regions, 50 studies utilized brain datasets, and 10 studies used cardiac datasets (see Figure 5E), with a higher usage tendency for brain and cardiac datasets, as calculated by linear regression.

### 6.2. Design

#### 1. Deep learning library:.

**Figure 6:**
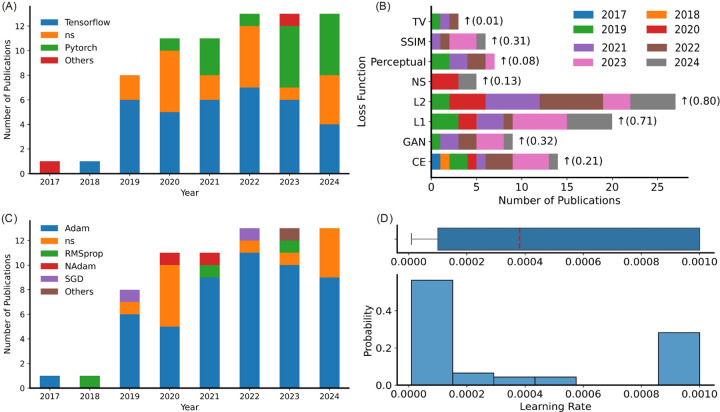
Neural network design traits. (A) DL libraries, (B) loss functions, and (C) Optimizers used by different studies. The arrow indicate the trend of the variable usage over time. The number inside the parenthesis represents the slop value calculated using logistic regression. (D) Distribution of the learning rate used by the studies over time and the red dashed line represents the mean value.

TensorFlow [167] was the most commonly used library, employed in 35 studies. PyTorch [168] was the second most used, with 19 studies. MATLAB and Keras (without TensorFlow as the backend) were each used in only one study. Logistic regression analysis indicates a higher tendency toward the use of PyTorch compared to TensorFlow, as illustrated in Figure 6A.

#### 2. Loss function:.

The loss or cost functions used in the studies are depicted in Figure 6B and summarized in Table 3. L2 and L1 loss functions were the most frequently used, utilized by 26 and 25 studies, respectively [75, 63, 87, 10]. Cross entropy, used in 14 studies, ranked second in usage. The L1 and L2 loss functions were predominantly used by the MoCo models, and cross-entropy was used by the MoDe models [169, 5, 170, 93].

While the L2 loss function is computationally inexpensive, it often results in overly smoothed images because it penalizes large errors more severely due to its squared term. In contrast, the L1 loss, which is also computationally efficient, was employed by a many studies and is know to produce sharper images compared to L2 loss. To improve the perceptual quality of reconstructed images, Perceptual loss has been utilized by minimizing the difference in image embeddings derived from pretrained networks such as VGG16 [127], though this approach is computationally expensive. The structural similarity index (SSIM) [171] loss function was also utilized as a less computationally intensive method to preserve perceptual quality [97]. Despite their advantages, both Perceptual and SSIM losses might be less sensitive to fine details, necessitating the use of additional L1 or L2 loss functions [99, 92].

#### 3. Optimizer:.

The optimizers used by the studies over time are illustrated in Figure 6C. The Adam optimizer [172] was the most commonly used, employed in 50 studies. Linear regression analysis indicates a positive tendency in the increasing popularity of the Adam optimizer over time. Additionally, the histogram of learning rate values used by the studies is illustrated in Figure 6D, with the red line indicating a mean learning rate value of 4×10^−4^. We reported the initial learning rate values when a study used learning rate schedulers to modify them. The purpose of Figure 6D is not to compare learning rates across different architectures and optimizers, but rather to provide an overview of the distribution of initial learning rates reported in the literature, thereby reflecting common practices in hyperparameter selection. In contrast, SGD and AdamW optimizers were used less frequently.

### 6.3. Evaluation metrics and performance

We also reported the evaluation metrics that were reported by the studies. Quantitative metrics were typically reported for *in-silico* datasets where ground truth motion-free images are available. However, when ground truth data is not available, raters graded image quality for motion-corrected brain images [110] and cardiovascular images [173]. This method is subjective and is better used in conjunction with quantitative results [72].

**Figure 7:**
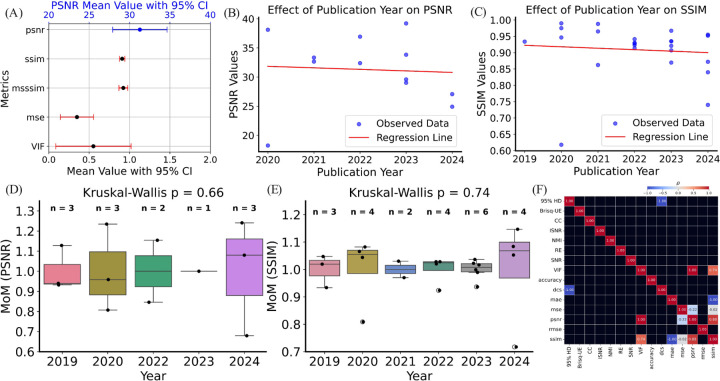
Performance improvement relative to the average values of metrics. (A) Average values with 95% CI are given for PSNR, SSIM, MS-SSIM, MSE, and VIF. These metrics were selected because they appeared more than three times across the reviewed studies. Due to the different value ranges, the PSNR axis is shown separately on the top in blue. (B) and (C) illustrate meta regression results for PSNR and SSIM to assess whether publication year accounts for variance in these metrics; we fit ordinary least squares meta regression with publication year as the predictor and PSNR or SSIM as the response and report heteroskedasticity robust standard errors (Section 5.2). (D) MoM of PSNR over time is illustrated. The numbers above the box plots indicate the number of studies published in that year. (E) MoM of SSIM over time. (F) Pearson correlation $\left(\rho \right)$ heatmap between pairs of metrics. Black regions indicate where ρ values are not available due to small sample size. MoM is defined as the study value divided by the corpus wide mean of that metric.

Figure 7A presents a comparison of the mean values and 95% confidence intervals for various image quality metrics, including PSNR, SSIM, multi-scale structural similarity index (MS-SSIM), mean squared error (MSE), and visual information fidelity (VIF). The metrics are plotted on the y-axis, with their mean values with associated confidence intervals displayed on the x-axis: downward SSIM, MS-SSIM, MSE, and VIF metrics, and upward for the PSNR metric. PSNR is highlighted as the primary metric, with a mean value centered around 30 dB, which is consistent with the typical values reported in the literature. The narrow confidence intervals for PSNR indicate a relatively low variability among the reported values, whereas other metrics like MSE exhibit wider confidence intervals, suggesting greater variability.

The statistical analysis for the trend of PSNR values over publication years, as depicted in Figure 7B, reveals an R^2^ value of 0.004, indicating that only 0.4% of the variance in PSNR values can be explained by the publication year. The F-statistic of 0.03831, with an associated *p*-value of 0.849, confirms that the regression model is not statistically significant, suggesting that there is no significant linear relationship between publication year and PSNR values in the observed data. The results imply that, despite the slight downward trend observed in the scatter plot, publication year does not have a meaningful impact on PSNR values. The same trend is observed for SSIM. The statistical analysis for SSIM values over publication years (see Figure 7C) shows an R^2^ value of 0.007, suggesting that only 0.7% of the variance in SSIM values is explained by the publication year. The F-statistic of 0.1520 and the corresponding *p*-value of 0.700 also indicate that the regression model is not statistically significant. Therefore, the slight downward trend observed in the SSIM values over time is not statistically supported, and publication year does not significantly affect SSIM values.

Figure 7D presents a boxplot of PSNR MoM across different publication years. These MoMs are compared across years to assess whether the quality improvement, as measured by PSNR, has remained consistent. The Kruskal-Wallis test yielded a *p*-value of 0.66, suggesting no significant difference in the PSNR MoMs across the years. This indicates that, despite some year-to-year variations, the overall trend in PSNR improvements has remained stable. Figure 7E shows a similar boxplot for SSIM MoM across the same publication years. The Kruskal-Wallis test resulted in a *p*-value of 0.74, indicating no significant differences in SSIM MoM over the years. Like PSNR, this suggests that SSIM metric values have not experienced significant fluctuations over time.

Figure 7F provides a heatmap representing the correlation matrix (ρ values) between various image quality metrics. The matrix illustrates how strongly different metrics are correlated with each other. Strong positive correlations are indicated by darker red shades, while weaker correlations or negative correlations are in lighter shades. For example, PSNR shows a strong correlation with SSIM and other related metrics, while MSE, which is inversely related to PSNR, shows an expected negative correlation. This matrix is critical for understanding the interdependence of different image quality metrics and how they collectively influence the overall assessment of image quality (see Table 4).

## 7. Discussion

DL algorithms have markedly transformed the landscape of MRI motion correction and detection, offering substantial improvements in correcting and detecting motion artifacts. We performed a systematic review and meta-analysis to highlight the motion detection and correction techniques, progress, trends of data usage and DL models, limitations and challenges, future directions of DL-based motion detection (MoDe) and motion correction (MoCo) models.

### 7.1. Advancements and impact

One of the key advancements is the application of generative models like generative adversarial networks (GANs) and denoising diffusion probabilistic models (DDPMs) in this domain. These models have been adapted from their original tasks in image generation to handle effectively the unique challenges posed by MRI motion artifacts. GANs, for example, have been employed both for direct motion correction and as adversarial regularizers to enhance model performance, while DDPMs offer a novel way to reconstruct high-quality images from corrupted data. Additionally, the development of unsupervised and unpaired learning methods has reduced the dependency on large, paired datasets, which are often difficult and expensive to acquire.

The impact of these advancements is substantial, offering the potential for more accurate diagnostics, fewer repeated scans, and overall improvements in patient care. By enhancing image quality and diagnostic accuracy, DL-based MoCo and MoDe models not only improve clinical outcomes but also contribute to cost savings in healthcare settings. The ability to generalize these models across different MRI sequences and patient populations further underscores their robustness and applicability in real-world clinical scenarios. As these technologies continue to evolve, they are poised to become integral tools in the ongoing effort to improve the reliability and accessibility of MRI diagnostics.

### 7.2. Challenges and future direction

Although DL-based MoCo and MoDe techniques have achieved excellent results and outperformed traditional methods [34], they require large datasets with raw k-space data for training. While large-scale 2D raw k-space datasets such as fastMRI [140] are already available and have greatly advanced the field (e.g., in compressed sensing MRI [135]), there remains a critical need for large 3D MRI datasets. Releasing such 3D datasets could similarly transform the development and benchmarking of DL-based MoCo and MoDe methods. Moreover, zero-shot and transfer learning methods can be explored to detect and remove motion artifacts. Zero-shot learning models can be designed to handle motion artifacts in MRI scans without being explicitly trained on every possible type of motion artifact. As such, zero-shot learning leverages semantic information or prior knowledge about motion artifacts to make accurate corrections and detections even for types of motion artifacts that the model has not encountered during training.

On the other hand, in the transfer learning technique, a model is first trained on a large dataset, such as a natural image dataset or a medical imaging dataset that captures related features. Once pre-trained, the model is fine-tuned on a smaller domain-specific dataset containing MRI scans with motion artifacts. During this fine-tuning phase, the model’s parameters are adjusted to specialize in detecting and correcting the motion artifacts present in the MRI data. Transfer learning is particularly effective for MoCo and MoDe tasks because it allows the model to build on the knowledge gained from other tasks, which share underlying principles with motion detection and correction. This approach can markedly reduce the amount of paired data needed for training while improving the model’s ability to generalize to new, unseen motion artifacts.

Another issue with DL techniques is their generalization to different MRI sequences and datasets. While we were unable to find studies specifically exploring the generalizability of these models to out-of-distribution datasets, it is hypothesized that their performance may decline when applied to such datasets. We further speculate that the use of vision-language models [174] could enhance generalization, as text prompts might provide additional context that can guide the motion detection and correction processes, potentially making these models more robust across varied datasets and MRI sequences.

A further challenge arises from the heterogeneity of reported architectures. Many studies employ hybrid models that combine elements such as convolutional backbones, Transformer blocks [175], GAN frameworks, or diffusion modules. This blending of design paradigms reflects the current direction of the field, but it makes it difficult to categorize studies unambiguously or to disentangle the contribution of each component. As a result, comparative analysis across architectures is limited, underscoring the need for standardized benchmarks and reporting practices in future work.

Our systematic review and meta-analysis focused on how much DL models improved image quality in MoCo tasks and their performances in MoDe tasks. However, such a comparison is challenging as the majority of studies used motion-simulated *in-silico* datasets, which may not be consistent in different studies due to the differences in the locations of motion-corrupted lines. Lines close to the k-space center contribute to the blurring and ghosting, while the peripheral lines contribute to ringing artifacts [87]. Standardized and reproducible methods are required to generate motion artifacts in different studies. One quick remedy could be the consistent reporting of quantitative metrics that can specify ghosting and blurring contents in motion-corrupted MR images such as PSNR and MS-SSIM metrics.

## 8. Conclusion

In this systematic review and meta-analysis, we explored the landscape of DL techniques for MoCo and MoDe in MRI. The advancements in DL have shown significant potential in addressing the challenges posed by motion artifacts in MRI. These models have demonstrated their potential to enhance image quality, reduce the need for repeated scans, and improve diagnostic accuracy, thereby contributing to better clinical outcomes and cost savings in healthcare.

However, the generalizability of these models across different MRI sequences and out-of-distribution datasets remains a concern. While the current research shows great promise, there is a clear need for further studies to explore the robustness of these models in varied clinical settings. Additionally, the integration of emerging techniques such as zero-shot learning and transfer learning could further enhance the ability of these models to generalize and perform effectively in diverse scenarios.

Overall, the continued evolution of DL-based MoCo and MoDe models is poised to play a crucial role in the future of MRI diagnostics, offering the potential to improve significantly the reliability and accessibility of high-quality medical imaging. As these technologies mature, their impact on patient care and healthcare systems will likely become even more profound.

## Figures and Tables

**Table 1:** Our criteria to collecting data for each study by modifying the CLAIM criteria are summarized.

Category	Item	Explanation
Data	Dataset	What dataset(s) were used and how was collected
	Region	Which region of the body was studied
	Ground truth	What were the ground truth, e.g. *in-silico* or *in-vivo*.
	MRI sequence	What MRI sequence(s) was included in the study (T_1_, T_2_, etc.)
	Motion simulation	What was the motion-simulation method and how the corresponding values were selected
	Partition	What was the partition strategy, percentage of training and testing partition data
	Availability	Whether data are publicly available or not
	Augmentation	What augmentation method was used, e.g. rotation, flipping, etc.
Model	Training	What was the training method, e.g. image-based MoCo, estimation-based MoCo, model-based MoCo, and MoDe
	Library	What library was used to implement a proposed DL model, e.g. PyTorch and TensorFlow
	Input domain	What was the input’s domain, k-space or image space
	Open source	Whether the original implementation is completely available, and not partially
	Loss	What was the loss function and how many were used
	Optimizer	What was the optimizer and the corresponding learning rate
Evaluation method	Metric	How many quantitative metrics were reported, and what were they, e.g. PSNR, SSIM, and NMSE
	Comparison	What other models were the proposed model compared with
	Motion levels	How many motion artifact levels were the model evaluated for, e.g., minor, moderate, and heavy
	Testing mode	What was the testing dataset, e.g. *in-vivo* and *in-silico*
	External dataset	Whether an external dataset was used to evaluate the proposed model’s generalization
	Diagnostic	Whether a clinician evaluated the diagnostic accuracy of the MoCo models
Discussion	Novelty	What could be the potential novelty of the proposed method
	Limitation	What are the main limitation of the study

**Table 2:** Public datasets used by the studies.

Dataset	Sample Size	Region	Used by
fastMRI [140]	8,400 scans	Brain, knee	[75, 10, 60, 70, 143, 144]
MR ART [141]	148 subjects	Brain	[10, 76, 72, 145, 8, 146]
HCP [147]	1,200 subjects	Brain	[63, 67, 70, 71, 148]
UK Biobank [149]	100,000 subjects	Whole body (focus on brain, heart)	[131, 132, 110, 114, 8]
IXI	600 subjects	Brain	[150, 93, 117, 146, 151]
ABIDE [152, 153]	2,226 subjects	Brain	[63, 154, 29, 155]
OSAIS [156]	565 subjects	Brain	[154, 131, 155]
ACDC [157]	150 patients	Heart	[99, 109, 148]
ADNI [158]	819 subjects	Brain	[87, 159]
GLIS-ART [160]	230	Brain	[76, 72]
MSSEG [161]	53 patients	Brain (Multiple Sclerosis)	[159]
MNI BITE [162]	14 subjects	Brain	[159]
IBSR [163]	18 subjects	Brain	[150]
Forstmann, et al. [164]	30 subjects	Brain	[65]
1000BRAINS [165]	1,000 subjects	Brain	[166]

**Table 3:** Summary of Loss Functions Used in AI for Motion Artifact Detection and Correction.

Loss Function	Mathematical Formula	Pros and Cons
Total Variation (TV)	*$\sum_{i,j}\sqrt{{\left(x_{i+1,j}-x_{i,j}\right)}^{2}+{\left(x_{i,j+1}-x_{i,j}\right)}^{2}}$ where x is the image, i, j are pixel indices*	**Pros:** Effective in reducing noise and smoothing motion artifacts while preserving edges, making it suitable for motion correction tasks. **Cons:** May oversmooth images, potentially eliminating fine motion details important for accurate detection and correction.
SSIM (Structural Similarity Index)	*$\frac{\left(2\mu_{x}\mu_{y}+c_{1}\right)\left(2\sigma_{xy}+c_{2}\right)}{\left(\mu_{x}^{2}+\mu_{y}^{2}+c_{1}\right)\left(\sigma_{x}^{2}+\sigma_{y}^{2}+c_{2}\right)}$ where μ is mean, σ is variance, $c_{1}$, $c_{2}$ are constants*	**Pros:** Preserves structural and perceptual quality, enhancing the integrity of corrected images in motion compensation. **Cons:** Computationally intensive and may be less sensitive to subtle motion artifacts, possibly overlooking minor corrections needed.
Perceptual Loss	*${\left\|\sum_{i}\phi_{i}\left(x\right)-\phi_{i}\left(y\right)\right\|}_{2}^{2}$ where $\phi_{i}$ is the feature map of the i-th layer of a pretrained network such as VGG16* [127]	**Pros:** Aligns corrected images with human perception by utilizing deep feature representations, improving motion correction quality. **Cons:** Depends on pretrained models not specialized for motion correction, and increases computational load due to deep network processing.
L2 Loss	*${\left\|x-y\right\|}_{2}^{2}$ where x is the predicted image, y is thetarget image*	**Pros:** Simple to implement with smooth gradients, facilitating the training of models for motion correction. **Cons:** Over-penalizes large errors from motion artifacts, potentially leading to blurred images and loss of important details.
L1 Loss	*${\left\|x-y\right\|}_{1}$ where x is the predicted image, y is the target image*	**Pros:** More robust to outliers introduced by motion, better at preserving sharp edges during correction. **Cons:** May produce less smooth results, leaving residual artifacts after motion correction.
GAN Loss	*$E_{x}\left[\log D\left(x\right)\right]+E_{z}\left[\log\left(1-D\left(G\left(z\right)\right)\right)\right]$ where G is the generator, D is the discriminator, x is real data, z is noise*	**Pros:** Generates highly realistic images by learning the distribution of motion-free data, effectively correcting complex motion artifacts. **Cons:** Training can be unstable and prone to mode collapse, requiring careful tuning and increasing complexity in motion correction models.
Cross Entropy Loss	*$-\sum_{i}p\left(i\right)\log q\left(i\right)$ where p is the true distribution, q is the predicted distribution*	**Pros:** Effective for motion detection when framed as a classification problem, distinguishing between motion and motion free regions. **Cons:** Sensitive to class imbalance common in motion detection tasks, potentially leading to biased detection models.

**Table 4:** Summary of Image Quality Metrics for Motion Artifact Correction and Detection

Metric	Formula	Comment
MAE	$\frac{1}{n}\sum_{i=1}^{n}\left|x_{i}-y_{i}\right|$	Measures the average absolute difference between the artifact-corrected image and the ground truth; simple to compute but may not capture perceptual differences introduced by motion artifacts.
MSE	$\frac{1}{n}\sum_{i=1}^{n}{\left(x_{i}-y_{i}\right)}^{2}$	Calculates the average of squared differences; sensitive to large errors typical in regions affected by motion; useful for quantifying overall correction performance but may exaggerate the impact of outliers.
PSNR	$10\log_{10}\left(\frac{{\text{MAX}}^{2}}{\text{MSE}}\right)$	Assesses the peak error between the corrected image and ground truth; higher PSNR indicates better artifact suppression; however, it may not align with perceived visual quality in the presence of motion artifacts.
SSIM	$\frac{\left(2\mu_{x}\mu_{y}+C_{1}\right)\left(2\sigma_{xy}+C_{2}\right)}{\left(\mu_{x}^{2}+\mu_{y}^{2}+C_{1}\right)\left(\sigma_{x}^{2}+\sigma_{y}^{2}+C_{2}\right)}$	Evaluates structural similarity, focusing on luminance, contrast, and structure; more sensitive to motion-induced distortions; better correlates with human perception of image quality after artifact correction.
MS-SSIM	$\prod_{j=1}^{M}{\text{SSIM}}_{j}{\left(x,y\right)}^{\alpha j}$	Multiscale extension of SSIM; assesses image quality across multiple resolutions; effectively captures the correction of motion artifacts occurring at different spatial scales.
Dice Score	$\frac{2\left|X\cap Y\right|}{\left|X\right|+\left|Y\right|}$	Used for evaluating segmentation accuracy in artifact detection; measures the overlap between detected motion artifact regions and the ground truth; sensitive to small artifacts and class imbalance in motion prevalence.

## Highlights

- Conducted the first meta-analysis of AI-driven MRI motion artifact detection and correction methods
- Systematically reviewed deep learning models used for MRI artifact correction
- Identified current dataset trends, key challenges, and existing research gaps
- Proposed directions to develop robust, generalizable, and effective AI models

## Abbreviations

B_0_: Magnetic field strength in MRI
CLAIM: Checklist for Artificial Intelligence in Medical Imaging
CNN: Convolutional Neural Network
CycleGAN: Cycle-Consistent Generative Adversarial Network
cGAN: Conditional Generative Adversarial Network
DDPM: Denoising Diffusion Probabilistic Model
DNN: Deep Neural Network
DL: Deep Learning
DVF: Deformation Vector Field
DWI: Diffusion Weighted Imaging
FLAIR: Fluid-Attenuated Inversion Recovery
GAN: Generative Adversarial Network
MRI: Magnetic Resonance Imaging
MoCo: Motion Correction
MoDe: Motion Detection
MoM: Multiple of the mean
MSE: Mean Squared Error
MS-SSIM: Multi-scale Structural Similarity Index
NMSE: Normalized Mean Squared Error
PE: Phase Encoding
PSNR: Peak Signal-to-Noise Ratio
SSIM: Structural Similarity Index
T_1_c: Postcontrast T_1_-weighted
T_1_w: T_1_-weighted
